# Somatostatin Receptors in Human Meningiomas—Clinicopathological Aspects

**DOI:** 10.3390/cancers13225704

**Published:** 2021-11-15

**Authors:** Sofie Eline Tollefsen, Anders H. Jarmund, Borgny Ytterhus, Øyvind Salvesen, Patricia Mjønes, Sverre Helge Torp

**Affiliations:** 1 Department of Clinical and Molecular Medicine, Faculty of Medicine and Health Sciences, Norwegian University of Science and Technology (NTNU), 7491 Trondheim, Norway; anders.h.jarmund@ntnu.no (A.H.J.); borgny.ytterhus@ntnu.no (B.Y.); oyvind.salvesen@ntnu.no (Ø.S.); patricia.mjones@ntnu.no (P.M.); sverre.torp@ntnu.no (S.H.T.); 2 Centre of Molecular Inflammation Research (CEMIR), Norwegian University of Science and Technology (NTNU), 7491 Trondheim, Norway; 3 Department of Pathology, St. Olavs Hospital, 7006 Trondheim, Norway

**Keywords:** brain tumors, meningioma, immunohistochemistry, somatostatin receptors, SSTR, digital pathology, diagnosis, prognosis

## Abstract

**Simple Summary:**

Meningioma diagnostics and grading are currently based on subjective histopathological criteria given by the 2016 World Health Organization (WHO) classification. However, biomarkers may provide a more objective approach to diagnostics. This study was designed to elucidate the diagnostic and prognostic value of somatostatin receptors (SSTRs) as biomarkers in meningiomas, which could help to identify patients with a higher risk of recurrence and provide more personalized treatment. We have confirmed, in a population of 162 patients, that SSTRs have diagnostic value and may aid in the differentiation between WHO grade 1 and grade 2 tumors. Furthermore, SSTR1, SSTR2 and SSTR5 were associated with higher malignancy grades. SSTR2 expression was found to be characteristic in meningiomas. To maintain objectiveness, we scoped for a digital evaluation of immunoreactivity. We aim to impact and motivate researchers to further investigations towards more objective criteria in meningioma diagnostics, which in turn will improve patient care.

**Abstract:**

Meningiomas have high recurrence rates despite frequently benign histopathological appearances. Somatostatin receptors (SSTRs) may be reliable biomarkers that could identify patients with increased risk of recurrence. Even though SSTRs are previously detected in meningiomas, their associations to clinicopathological features remain unclear. The aim of this study was to investigate the diagnostic and prognostic value of SSTRs in a large series of human meningiomas with long follow-up data. Immunohistochemistry was used to measure the expression of SSTR1-SSTR5 in tissue samples from 162 patients diagnosed with intracranial meningiomas of World Health Organization (WHO) grade 1 or 2. Digital scoring and a manual staining index were applied to assess immunoreactivity. All SSTRs, except SSTR4, were upregulated in our series of meningiomas. SSTR1 (*p* = 0.036), SSTR2 (*p* = 0.036) and SSTR5 (*p* = 0.029) were associated with a higher malignancy grade. SSTR2 presented as the most reliable marker. Only SSTR2 was associated with time to recurrence (TTR) in univariate Cox regression analyses. Manual staining index was strongly correlated with digital scoring for all SSTRs (*r* > 0.65, *p* < 0.001). SSTRs, and especially SSTR2, are useful in the diagnostics of meningiomas, even though their prognostic value appears limited. Digital scoring is valuable to ensure reproducibility.

## 1. Introduction

Meningiomas are typically benign and slow-growing neoplasms, yet a substantial number of patients experience tumor recurrence [1,2,3,4]. Identifying patients with increased risk for recurrence is important to provide appropriate care, especially since the current treatment options for meningiomas leave the patients vulnerable to severe complications [4,5,6]. The histopathological classification given by the World Health Organization (WHO) is used to design personalized treatment, but the current 2016 WHO classification for meningiomas is suboptimal due to its subjective histopathological criteria and troublesome reproducibility [2,7]. Accordingly, it is necessary to find objective biomarkers that can identify patients with a higher risk for recurrence.

Somatostatin receptors (SSTRs) are present in many tumors, including meningiomas [8,9,10], and may constitute diagnostic and prognostic biomarkers due to their involvement in tumorigenesis. SSTRs comprise a family of five (SSTR1-SSTR5) G-protein-coupled receptors that bind to the regulatory peptide somatostatin and its analogs to regulate cell growth, inhibit proliferation and angiogenesis, and promote apoptosis in both normal and neoplastic cells [11,12,13,14,15,16].

SSTR2 is considered a reliable diagnostic biomarker for meningiomas [17] and is the most prevailing SSTRs in human meningiomas [9,10,18,19]. The attempts to relate SSTR2 to tumor grade have been unsuccessful [9,14,18,20], despite the findings of higher SSTR2 expression in meningothelial tumors [14,20] and in tumors with high microvascular density [21]. Other SSTRs have also been detected in meningiomas, and a higher expression of SSTR5 was found in WHO grade 2 meningiomas [10], but their relationship to histopathological features and tumor grade are sparsely described [9,10,18,19].

Digital pathology is increasingly used in quantification of biomarkers, much due to the enhanced use of whole slide scanning and improved software. Compared to the conventional microscopy of immunohistochemical reactivity, digital tools may provide several benefits, such as increased reproducibility, objectivity and accuracy [22]. To our knowledge, no previous studies have used digital tools to determine the immunohistochemical expression of SSTRs in meningiomas.

The aim of this paper was to use both conventional microscopy and digital tools to investigate the immunohistochemical expression of SSTRs in a large series of human WHO grade 1 and 2 meningiomas and relate these findings to histopathology, tumor grade and prognosis.

## 2. Materials and Methods

### 2.1. Patients

The inclusion of 196 patients and the collection of clinical data has previously been reported [23,24]. All patients were >18 years old and operated for primary intracranial meningioma in the period of 1 January, 1991 to 31 December, 2000 at St. Olavs hospital, Trondheim University Hospital, Trondheim, Norway. Due to a low number of WHO grade 3 tumors, this study only includes 162 WHO grade 1 and 2 tumors with enough tissue for tissue microarray (TMA) preparation, as previously conducted by Arnli et al. [25]. The 162 tumors were reviewed according to the 2016 WHO classification [26]. Patients were followed until death or for a maximum of 18 years. The end of follow-up was 1 January, 2009. Median follow-up time was 11 years.

Additional tissue samples were collected to establish the SSTRs expression in adjacent meninges and the following differential diagnoses: neurofibroma, schwannoma, solitary fibrous tumor/hemangiopericytoma and hemangioblastoma. Two formalin-fixed, paraffin-embedded tissue specimens of each differential diagnosis and ten adjacent meninges to tumor tissue were obtained.

### 2.2. Immunohistochemistry

Paraffin sections of 4 micrometers were dried overnight at 37 °C and then for one hour at 60 °C before deparaffinization and rehydration. Heat induced epitope retrieval (HIER) was performed in target retrieval solution (TRS) pH6 for 20 min at 97 °C and then cooled to 65 °C, conducted by PT Link (DAKO Denmark A/S, Produktionsvej 42 DK-2600 Glostrup, Denmark). DAKO Autostainer Plus was used for immunodetection. After incubation of primary antibodies, sections were treated for 10 min in Dako REAL Peroxidase Blocking Solution (S2023) to prevent endogenous peroxidase activity. Secondary antibodies were incubated for 30 min (HRP Rabbit/Mouse EnVision—Polymer, Dako REAL Envision Detection System K 5007). DAB+ Chromogen (Dako REAL Envision Detection System) was utilized for 10 min before conducting hematoxylin counterstain. Lastly, sections were dehydrated and coverslips applied. Positive controls of duodenum, stomach, pancreas and pituitary gland were treated in the same manner as tumor samples. The primary antibodies were omitted for negative controls. Details on antibodies are listed in Table 1. TMA cores with <50% remaining tissue were excluded.

### 2.3. Manual Scoring of Immunohistochemistry

Each tumor was given a staining index (SI) by an experienced neuropathologist (S.H.T.) as part of the manual evaluation of immunohistochemistry (IHC). SI was defined as the product of percentage of positive cells and staining intensity. Percentage of positive cells was scored as <10% (1), 10–50% (2) or >50% (3), while intensity was scored as negative (0), weak (1), moderate (2) or strong (3) [27]. One collective SI was estimated for all three TMA cores from each patient.

The immunoreactivity of differential diagnoses and meninges was manually assessed and quantified as positive or negative.

### 2.4. Image Acquisition and Digital Evaluation of SSTRs Expression

A digital evaluation of SSTRs expression was conducted as suggested by Varghese et al. [28] with adjustments. Each TMA core had a diameter of 1000 µm and were constructed from areas with only tumor tissue and without hemorrhage or necrosis. All TMA cores were scanned using Olympus VS120S5 with a ×20 objective lens and the resulting images were processed in ImageJ, version 1.53c. Color deconvolution is a technique to transform the ordinary color channels of an image (such as red, green, and blue) to channels representing specified colors [29]. In ImageJ, color deconvolution was applied with “H DAB” as vector so that a new image was generated where the pixel intensity reflects the intensity of H DAB in the corresponding pixel in the original image. Next, a histogram of pixels per intensity was created for each image (Supplementary File S1). The intensity of each pixel was measured on a scale from 0 (darkest shade of color) to 255 (lightest shade of color). The total number of pixels per sample was 24,304,900. To exclude background stain from the digital analysis, a threshold was preselected for each SSTR, where pixels with intensity values over the selected threshold predominantly constituted background stain and were omitted from the calculation of digital score (DS) and subsequent analysis. The thresholds were based on manual evaluation of five TMA cores from different patients and were as follows: 220 for SSTR1, 219 for SSTR2, 222 for SSTR3, 211 for SSTR4, and 221 for SSTR5. The remaining pixels were divided into four zones based on intensity, and each zone was assigned one of the following scores [28]: highly positive (intensities < 61, score 4), positive (intensities 61–120, score 3), low positive (intensities 121–180, score 2), and negative (intensities >180, score 1). A digital score (DS) was calculated as the sum of zone scores weighted by the proportion of pixels in the corresponding zone, as Formula (1):

(1) $$\mathrm{DS}=\left(4\cdot n_{Highly\;positive}+3\cdot n_{Positive}+2\cdot n_{Low\;positive}+n_{Negative}\right)/N_{Total}$$

where $N_{Total}$ is the total number of pixels and n the number of pixels in the corresponding zone [28]. Thus, DS ranges from 1 (negative pixels only) to 4 (highly positive pixels only). A single score was sustained for the three TMA cores of each patient. DSs were calculated in RStudio, version 4.0.2 (Supplementary File S2).

### 2.5. Statistical Analysis

Spearman’s rank-order correlation was applied to determine the correlation between DS and SI of each SSTR. The DS are continuous variables and may provide a more objective measure than SI. Hence, only the DS were used for further statistical analyses.

Mann-Whitney U test was used to compare the expression of SSTRs to the following dichotomic histological features: (1) 2016 WHO grade, (2) necrosis (yes/no), (3) hypercellularity (yes/no), (4) sheeting (yes/no), (5) macronuclei (yes/no), (6) small cells (yes/no) and (7) psammoma bodies (yes/no). Kruskal–Wallis test was applied to variables with more than two groups (tumor subtype), and if significant, followed by Dunn’s test and Holm–Bonferroni adjustment to preserve the familywise error rate. Tumor subtypes included meningothelial, fibrous, transitional and atypical tumors, as subtypes with only a single patient were excluded from the analysis.

Time to recurrence (TTR) and overall survival (OS) were used as endpoints for Cox regression analyses, where the digital SSTR scores were applied as continuous covariates. TTR was defined as time to recurrence or disease-related death [30].

Statistical analyses were conducted in IBM SPSS Statistics for Windows, Version 26.0. A *p*-value of <0.05 was considered significant.

## 3. Results

### 3.1. Patients

Among the 162 patients included, the median age at operation was 60 years (range: 25–86 years) and 73.5% (119 patients) were female (Table 2). In compliance with the 2016 WHO classification, 109 patients (67.3%) were diagnosed with grade 1 and 53 patients (32.7%) with grade 2. Most patients (84%) underwent a gross total resection (Simpson grade I–III) and were scored to WHO performance status 1 (68.7%). At follow-up, 41 patients had suffered from recurrence.

### 3.2. Immunohistochemical Expression of SSTRs in Meningiomas

The expression of SSTRs was assessed both manually and digitally (Table 3). Using manual staining index (SI), SSTR1 and SSTR2 presented with a median SI of 9, while SSTR3 and SSTR5 had a median SI of 6. SSTR4 had the weakest immunoreactivity (median SI = 0). With digital scoring (DS), SSTR1 (median DS = 2.72), SSTR2 (median DS = 2.24) and SSTR3 (median DS = 2.09) had the strongest immunoreactivity, while SSTR4 (median DS = 1.14) and SSTR5 (median DS = 1.51) had a weaker immunoreactivity.

**Figure 1:**
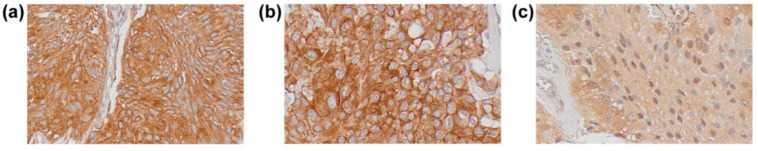
Different immunoreactivities demonstrated for SSTR1: (**a**) cytoplasmatic immunoreactivity; (**b**) membranous immunoreactivity; (**c**) nuclear immunoreactivity (×20).

**Figure 2:**
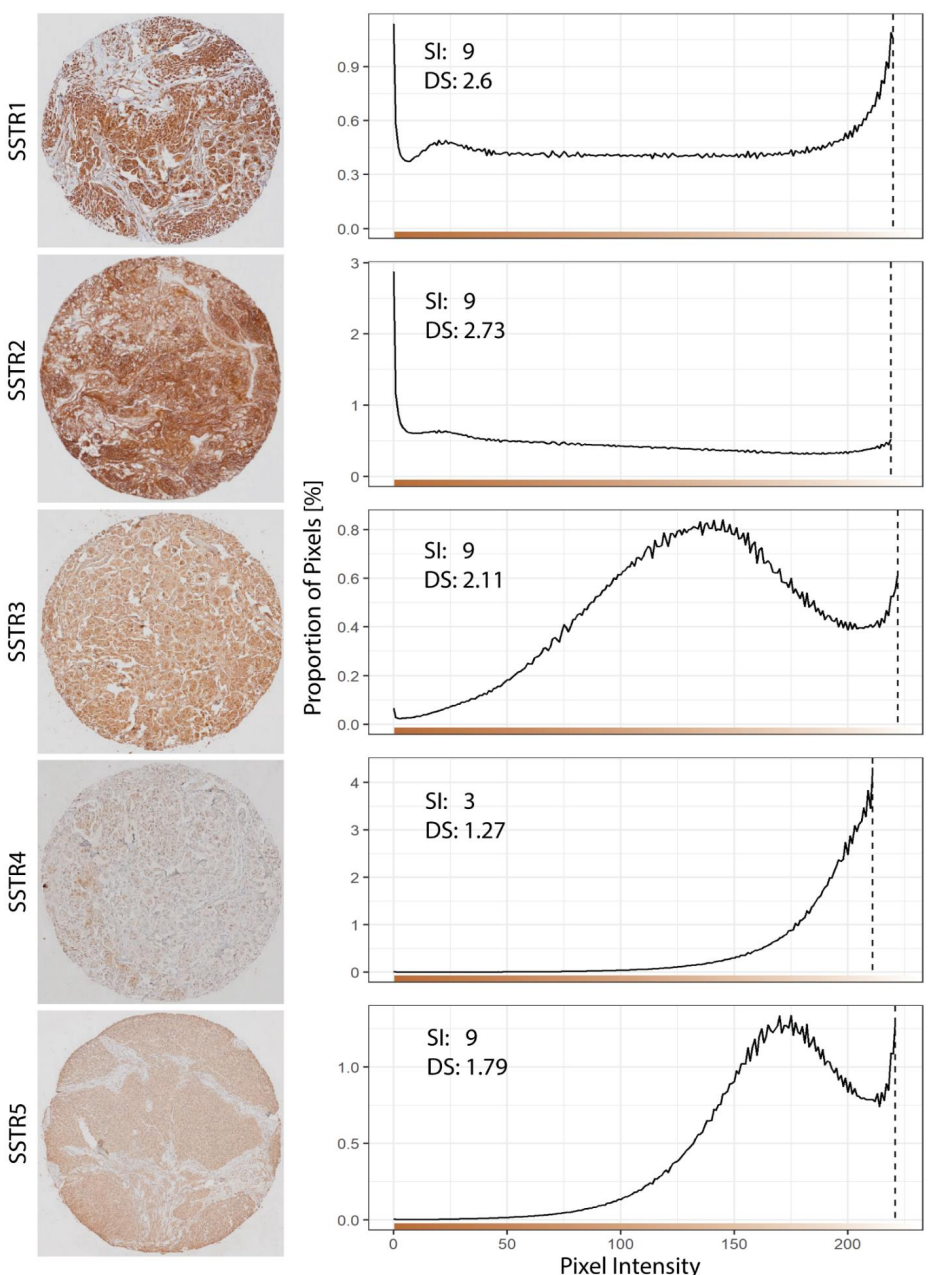
Representative tissue sections for each SSTRs (**left**) with their distribution of pixel intensity, SI and DS (**right**). A color scale of pixel intensity is included. Threshold for background stain was defined for each SSTRs as indicated by the dashed lines. Abbreviations: SI, staining index; DS, digital scoring (×20).

The expression pattern varied between the SSTRs (Figure 1 and Figure 2). SSTR1 and -2 had strong immunoreactivity, while SSTR3 and -5 exhibited a more moderate expression. In contrast, SSTR4 had sparse immunoreactivity. Cytoplasmatic immunoreactivity was observed for all SSTRs, while additional nuclear and membranous immunoreactivity was reported in some tumors for SSTR1 and -3. Also, SSTR4 occasionally expressed nuclear immunoreactivity. Regarding SSTR5, both nuclear and cytoplasmatic immunoreactivity were detected in all positive meningiomas. Fibroblasts were used as an internal negative control for all SSTRs. One tumor was excluded from further analysis due to lack of tissue after preparation.

The DS and SI showed good agreement according to Spearman’s rank-order correlation. The strong and positive correlation between DS and SI was highly significant for all SSTRs: SSTR1 (*r* = 0.657, *p* < 0.001), SSTR2 (*r* = 0.741, *p* < 0.001), SSTR3 (*r* = 0.745, *p* < 0.001), SSTR4 (*r* = 0.652, *p* < 0.001), and SSTR5 (*r* = 0.702, *p* < 0.001).

**Figure 3:**
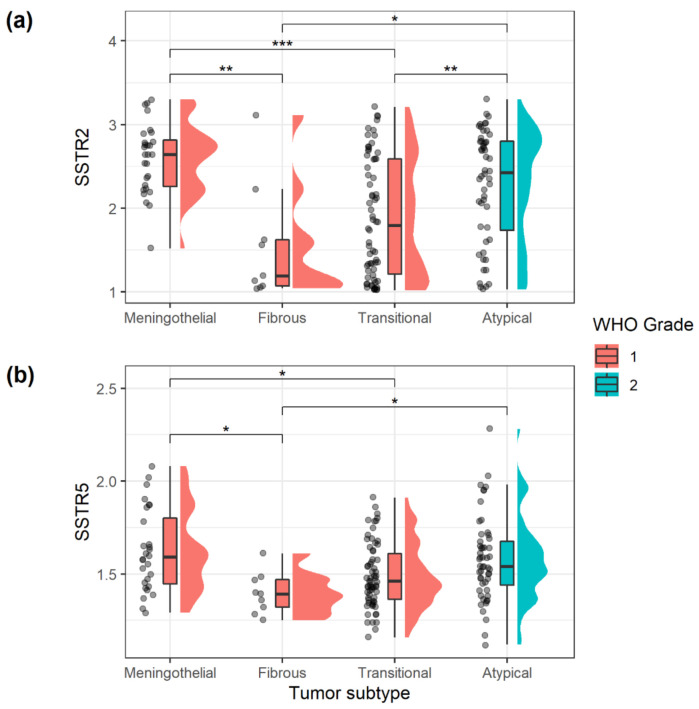
SSTRs expression related to tumor subtypes according to the digital scoring. Significant associations are marked: * *p* < 0.05, ** *p* < 0.01 and *** *p* < 0.001. Data are presented as boxplot (25th percentile, median and 75th percentile, whiskers 1.5 IQR), distribution and datapoints: (**a**) SSTR2 expression related to subtypes; (**b**) SSTR5 expression related to subtypes.

The SSTRs showed distinct expression in relation to specific histological features (Table 4). In general, SSTRs expression was stronger in WHO grade 2 than WHO grade 1 meningiomas, except for SSTR4 that showed similar expression in both grades. SSTR4 expression was, however, weaker in tumors with hypercellularity (*p* = 0.016). Differences related to tumor grade were evident for SSTR1 (*p* = 0.036), SSTR2 (*p* = 0.009) and SSTR5 (*p* = 0.029), while differences for SSTR3 (*p* = 0.059) were non-significant. SSTR2 expression was independently increased in the presence of necrosis (*p* = 0.010), in the presence of macronucleoli (*p* = 0.019) and with lack of psammoma bodies (*p* = 0.017). The expression of SSTR2 and SSTR5 varied significantly between tumor subtypes (*p* < 0.001 and *p* = 0.003, respectively) (Figure 3). Meningothelial meningioma WHO grade 1 and atypical meningioma WHO grade 2 showed significantly higher levels of SSTR2 than both transitional and fibrous subtypes (all Holm–Bonferroni adjusted pairwise *p*-values < 0.05). The differences were less prominent for SSTR5, but SSTR5 expression was higher in meningothelial tumors than in both fibrous (*p* = 0.019) and transitional tumors (*p* = 0.038), and higher in atypical tumors, compared to fibrous tumors (*p* = 0.037).

**Figure 4:**
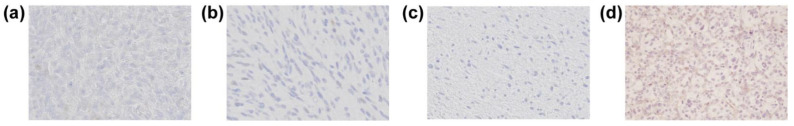
Differential diagnoses stained with SSTR2; (**a**) solitary fibrous tumor; (**b**) schwannoma; (**c**) neurofibroma; (**d**) hemangioblastoma (×20).

The SSTRs showed variable specificity for meningiomas (Figure 4), and SSTR2 appeared to be the most specific. Two tissue samples of each of the following differential diagnoses were tested for SSTRs expression: neurofibroma, schwannoma, solitary fibrous tumor/hemangiopericytoma and hemangioblastoma. SSTR1 and SSTR5 presented the same immunoreactivity, as one case of neurofibroma and one case of schwannoma were negative and the other tumor samples were positive. Regarding SSTR2, one case of neurofibroma and all cases of schwannoma, solitary fibrous tumor/hemangiopericytoma and hemangioblastoma were negative, while the other case of neurofibroma was positive. SSTR3 were immunoreactive in all tumors, whereas SSTR4 were negative in all differential diagnoses.

Normal meningeal tissue was not immunoreactive for SSTR4 and SSTR5. For SSTR1 and SSTR2, eight specimens (80%) were negative, while two samples were weakly positive or difficult to evaluate. In contrast, nine specimens (90%) expressed immunoreactivity for SSTR3.

### 3.3. SSTRs and Prognosis in Meningiomas

**Figure 5:**
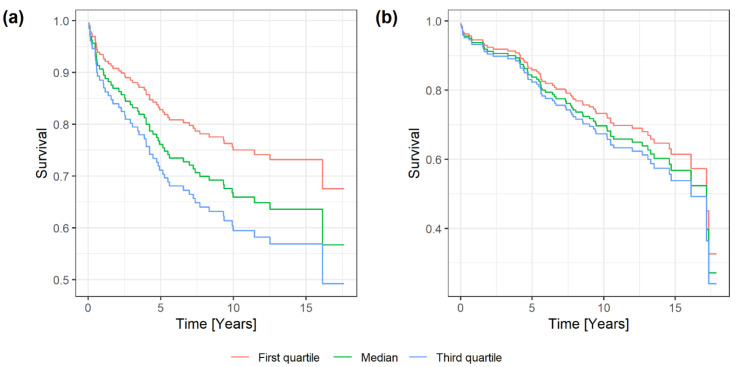
Univariate Cox regression survival plots for SSTR2, presented with 1st quartile, median and 3rd quartile; (**a**) SSTR2 related to time to recurrence (TTR); (**b**) SSTR2 related to overall survival (OS).

SSTRs expression was shown to have limited influence on OS and TTR (Table 5) in univariate cox regression analyses. Only SSTR2 expressed a significant association to TTR (*p* = 0.027). Survival plots for SSTR2, using cox regression, are presented in Figure 5.

## 4. Discussion

Although SSTRs have been considered as promising biomarkers for improved diagnostics of meningiomas, only a few studies have examined SSTR expression in relation to histopathology, tumor grade and prognosis. In this study, we found an overall high expression of SSTR1, -2 and -5 in human meningiomas with both manual and digital scoring. The three receptors were significantly more highly expressed in WHO grade 2 meningiomas than in WHO grade 1. SSTR3 was detected in tissues from all patients, whereas SSTR4 was predominantly negative. Only SSTR2 was significantly related to a higher prevalence of recurrence or disease-related death in univariate cox regression. We further found distinct expression of SSTRs in relation to atypical histopathological features, such as necrosis, macronucleoli and lack of psammoma bodies.

SSTR2 presents as an especially promising candidate for improving meningioma diagnostics. In accordance with previous immunohistochemical studies [9,10,17,31], the majority of meningiomas expressed SSTR2. The highly frequent expression of SSTR2 in meningiomas has also been confirmed by other techniques, such as reverse transcription polymerase chain reaction (RT-PCR), Western blot and Northern blot [9,18,19]. Most of the normal meningeal tissue and all tumor tissues from schwannomas, solitary fibrous tumors/hemangiopericytomas and hemangioblastomas were negative for SSTR2 in our study, supporting the high specificity for SSTR2 as a biomarker for meningiomas in diagnostic routine. This has also been confirmed by others [17,32].

The specificity of the other SSTRs appears more limited. SSTR1, -3 and -5 were expressed in most differential diagnoses. Hence, these receptors present low specificity for meningiomas. SSTR4 was not expressed in any of the differential diagnoses. Accordingly, this supports the abovementioned findings that SSTR2 is the most efficient diagnostic marker. Due to the low number of differential diagnoses in our study, further investigations are needed to confirm our findings.

The predominantly non-immunoreactive normal meningeal tissue supports a general upregulation of SSTR1 and -5 in most meningiomas. SSTR4 was also negative in all normal meningeal tissues and was only upregulated in a few tumors pointing to the minor role of this receptor in meningiomas. SSTR3 was present in both normal meningeal tissue and in all cases of meningiomas. Thus, SSTR3 may play a role both in normal and neoplastic meningeal tissue. Immunoreactivity was mostly cytoplasmatic, but some meningiomas also presented nuclear reactivity for SSTR1, -3, -4 and 5. This may be due to cellular internalization, where the SSTR-ligand complex translocates from the cytoplasm to the nucleus to utilize the effect of somatostatin [33].

Previous research has applied IHC, RT-PCR and Western blot to investigate the expression of SSTR1, -3, -4 and -5 with various results [9,10,18,19]. Non-coincident results may be due to different detection methods, differences in tissue preparation, and different antibodies, among others. If standardized, IHC represents an easily accessible and precise technique that could aid in further knowledge related to the diagnostic and prognostic value of SSTRs.

We report a significantly higher expression of SSTR1, -2 and -5 in grade 2 meningiomas, compared with WHO grade 1 tumors. SSTR3 showed a tendency of higher expression in WHO grade 2 tumors, but did not reach statistical significance. Silva et al. [10] also reported higher expression of SSTR5 in WHO grade 2 tumors. As for the other SSTRs, no previous studies have demonstrated a relationship to malignancy grade [9,18]. However, Barresi et al. [21] found a significantly higher microvessel density in SSTR2 positive cases than in those negative for SSTR2. This could suggest that SSTR expression is associated with neoangiogenic processes in meningiomas rather than to malignancy grade, as atypical and microcystic meningiomas are more vascularized with higher microvessel density than benign WHO grade 1 meningiomas [21].

Our study reports a significantly higher expression of SSTR2 in tumors with necrosis. SSTRs were reported to be associated with antiangiogenic effects in hepatocellular carcinoma [34] and, if there is a similar effect in meningiomas, necrosis could be caused by ischemia. Macronucleoli were also associated with a higher SSTR2 expression. SSTR4 displayed higher expression levels in tumors without hypercellularity, which could be due to the antiproliferative effect of SSTRs [15]. However, SSTR4 was weakly expressed, and its clinical relevance is uncertain. In our study, meningiomas without psammoma bodies had a significantly higher expression of SSTR2. The absence of psammoma bodies have previously been reported as a negative prognostic factor [23], and these results are in accordance with our finding of SSTR2 being more expressed in WHO grade 2 meningiomas.

In our series of meningiomas, the expression of SSTR2 and -5 was significantly different depending on meningioma subtype. This is also confirmed by Durand et al. [20] regarding SSTR2 mRNA. Two smaller studies found no significant relations between SSTR expression and tumor subtype [10,18], yet this could be caused by the lower statistical power. Meningioma subtypes have been shown to have different genetic alterations [2,35]. This may explain the differences in SSTR2 and -5 expression in relation to tumor subtype.

Regarding TTR and OS, our survival analyses were not significant for any of the SSTRs, except for SSTR2 related to TTR. This is in accordance with another study with median follow-up of only 18 months, which reports no relation between expression of SSTRs and tumor recurrence/regrowth [10]. These findings are in contrast to other tumors, such as gliomas [36], neuroblastomas [37] and pancreatic neuroendocrine tumors [38,39], in which SSTR2 has been reported as a positive prognostic marker. Accordingly, our findings indicate that the SSTR expression profile plays a minor role as a prognostic marker for meningiomas.

The digital evaluation of IHC provides a tool for objective and reproducible scoring with a continuous scale. Increasingly more pathology departments scan their tissue sections routinely, making the digital evaluation of IHC more accessible. As the manual evaluation of IHC is often both time-consuming and subject to visual bias, Varghese et al. [28] developed a method for quantitative evaluation and scoring of IHC, which our study adapted to better fit the immunostainings of SSTRs. The automated digital evaluation of IHC emphasizes all intensities of immunoreactivity equally to calculate a score, whilst SI accounts more for the stronger regions of immunoreactivity. The manual SI evaluation may easily exclude artifacts, which might be troublesome to exclude in the automated digital evaluation without applying artificial intelligence. Hence, the scoring systems differ from each other to some extent. To establish diagnostic cut-off values, further studies are needed. Concerning research, a standardized digital approach simplifies the comparison of research results across studies.

In terms of clinical value, SSTRs may be valuable for both the systemic treatment and imaging of meningiomas. At a cellular level, activation of SSTR1, -2 and -5 induces phosphotyrosine phosphatase (PTPη), which inhibits ERK1/2 phosphorylation, resulting in cell cycle arrest [12], and SSTR3 may induce apoptosis, as the tumor suppressor protein p53 is activated and increases the proapoptotic protein, Bax [16,40]. Somatostatin analogs are believed to have anti-tumor effects, even though results from case reports and smaller studies are inconclusive [41,42,43,44,45]. Currently, the European Association of Neuro-Oncology considers any pharmacotherapy for patients with meningioma to be experimental [35]. Our findings of high SSTR expression, except of SSTR4, support the presence of SSTRs as potential target points for pharmacotherapy. As somatostatin analogs present with different affinities depending on the SSTR [9,16,18,46], an individual mapping of SSTR expression prior to treatment could be beneficial for treatment response. Randomized controlled trials are needed to fully elucidate the effect of somatostatin analogs. As for imaging, meningothelial tissue may be differentiated from normal tissue using PET tracers, such as ⁹⁰Y-Dotatoc or ⁶⁸Ga-Dotatate that bind to SSTR2 [35,47,48].

The strength of this study is the large and population-based material with a long follow-up period. To our knowledge, no other studies have used digital pathology to provide an objective measurement of the immunohistochemical expression of SSTRs in meningiomas. Potential weaknesses are the retrospective nature of the study and the inherent challenges of immunohistochemistry.

## 5. Conclusions

In conclusion, all SSTRs, except SSTR4, are highly upregulated in meningiomas, supporting a crucial role of these receptors in the tumorigenesis of these tumors. Even though the prognostic value of SSTRs appears to be limited, they can be used as diagnostic markers for meningiomas, especially SSTR2, and can be valuable in any targeted clinical managements.

## Figures and Tables

**Table 1:** Antibodies.

SSTR1	Anti-SSTR1 (polyclonal IgG (rabbit), pH 6, 1:200 dilution, overnight incubation, somatostatin receptor 1, cat#HPA031506, Atlas Antibodies)
SSTR2	Anti-Somatostatin Receptor 2 antibody (UMB1)—C-terminal (monoclonal (rabbit), pH 6, 1:100 dilution, overnight incubation, somatostatin receptor 2, cat#ab134152, Abcam)
SSTR3	Somatostatin R3/SSTR3 Antibody (polyclonal (rabbit), pH6, 1:1000 dilution, overnight incubation, somatostatin receptor 3, cat#NB100-74538, Novus Biologicals)
SSTR4	Anti-SSTR4 (polyclonal IgG (rabbit), pH 6, 1:25 dilution, overnight incubation, somatostatin receptor 4, cat#HPA064252, Atlas Antibodies)
SSTR5	SSTR5: Anti-Somatostatin Receptor Type 5 Antibody (polyclonal (rabbit), pH 6, 1:100 dilution, overnight incubation, somatostatin receptor 5, cat#ab9287, Chemicon)

**Table 2:** Patient data.

Patient Data	WHO Grade 1	WHO Grade 2	All Grades
Total number, *n*	109	53	162
Median age at operation (range), years	58 (27–84)	65 (25–86)	60 (25–86)
Sex (female/male), *n*	84/25	35/18	119/43
WHO Performance Status (0–5), *n*	15/73/20/1/0/0	5/39/7/1/1/0	20/112/27/2/1/0
Simpson grade (I–V), *n*	30/47/13/19/0	11/26/9/7/0	41/73/22/26/0
Recurrence (yes/no), *n*	23/86	18/35	41/121

**Table 3:** Digital scoring and staining index of immunohistochemical expression of SSTRs in human meningiomas. The digital score goes from 1 to 4, whereas the staining index goes from 0 to 9.

SSTR	Digital Scoring		Staining Index	
	Mean (SD)	Median (Min.–Max.)	Mean (SD)	Median (Min.–Max.)
SSTR1	2.71 (0.43)	2.72 (1.33–3.55)	8.30 (1.41)	9 (2–9)
SSTR2	2.13 (0.71)	2.24 (1.02–3.30)	7.91 (2.02)	9 (2–9)
SSTR3	2.08 (0.23)	2.09 (1.58–2.62)	6.73 (1.83)	6 (3–9)
SSTR4	1.17 (0.10)	1.14 (1.04–1.65)	1.35 (1.65)	0 (0.00–6)
SSTR5	1.54 (0.21)	1.51 (1.12–2.28)	5.44 (1.81)	6 (3–9)

**Table 4:** SSTRs expression, using digital scoring, in relation to dichotomic histological features. *p*-values from two-tailed exact values Mann-Whitney U test with significant results in bold.

Categorical Variables	*n*	SSTR1		SSTR2		SSTR3		SSTR4		SSTR5	
Mann–Whitney U	161	Median (Range)	*p*-Value	Median (Range)	*p*-Value	Median (Range)	*p*-Value	Median (Range)	*p*-Value	Median (Range)	*p*-Value
WHO Grade 1	109	2.70 (1.33–3.55)	**0.036**	2.14 (1.02–3.30)	**0.009**	2.05 (1.58–2.58)	0.059	1.15 (1.04–1.65)	0.166	1.48 (1.12–2.08)	**0.029**
WHO Grade 2	52	2.82 (2.05–3.55)		2.52 (1.03–3.30)		2.12 (1.62–2.62)		1.12 (1.04–1.39)		1.55 (1.17–2.28)	
Necrosis present	37	2.76 (1.96–3.33)	0.647	2.61 (1.03–3.11)	**0.010**	2.12 (1.74–2.62)	0.285	1.13 (1.04–1.42)	0.368	1.54 (1.16–2.03)	0.054
Necrosis not seen	124	2.71 (1.33–3.55)		2.13 (1.02–3.30)		2.07 (1.58–2.58)		1.14 (1.04–1.65)		1.48 (1.12–2.28)	
Hypercellularity	42	2.68 (1.86–3.52)	0.686	1.94 (1.02–3.30)	0.208	2.10 (1.62–2.62)	0.664	1.12 (1.04–1.26)	**0.016**	1.50 (1.17–2.28)	0.975
Hypercellularity not seen	119	2.72 (1.33–3.55)		2.24 (1.03–3.30)		2.08 (1.58–2.58)		1.15 (1.04–1.65)		1.51 (1.12–2.08)	
Sheeting present	12	2.56 (2.22–3.37)	0.181	2.40 (1.09–3.17)	0.288	2.11 (1.77–2.42)	0.748	1.11 (1.05–1.23)	0.073	1.52 (1.25–1.95)	0.857
Sheeting not seen	149	2.73 (1.33–3.55)		2.19 (1.02–3.30)		2.08 (1.58–2.62)		1.15 (1.04–1.65)		1.50 (1.12–2.28)	
Macronucleoli present	11	2.83 (2.03–3.21)	0.743	2.84 (1.06–3.10)	**0.019**	2.11 (1.92–2.41)	0.486	1.11 (1.06–1.29)	0.418	1.61 (1.36–1.95)	0.051
Macronucleoli not seen	150	2.71 (1.33–3.55)		2.16 (1.02–3.30)		2.08 (1.58–2.62)		1.14 (1.04–1.65)		1.50 (1.12–2.28)	
Small cells present	15	2.87 (2.22–3.33)	0.054	1.86 (1.04–3.13)	0.803	2.10 (1.74–2.62)	0.355	1.11 (1.04–1.24)	0.181	1.50 (1.25–1.98)	0.526
Small cells not seen	146	2.70 (1.33–3.55)		2.24 (1.02–3.30)		2.09 (1.58–2.58)		1.14 (1.04–1.65)		1.51 (1.12–2.28)	
Psammoma bodies present	108	2.70 (1.58–3.42)	0.075	2.13 (1.02–3.30)	**0.017**	2.09 (1.61–2.62)	0.757	1.13 (1.04–1.65)	0.079	1.48 (1.12–2.03)	0.114
Psammoma bodies not seen	53	2.80 (1.33–3.55)		2.38 (1.03–3.30)		2.09 (1.58–2.55)		1.16 (1.04–1.52)		1.54 (1.17–2.28)	

**Table 5:** Association between SSTRs expression and overall survival (OS) or recurrence (TTR) from univariate Cox regression analyses. Significant results marked in bold.

SSTR	Measure	TTR (*n* = 55)	OS (*n* = 63)
SSTR1	HR (CI) (*p*-value)	1.012 (0.535–1.915) (0.972)	1.187 (0.640–2.203) (0.587)
SSTR2	HR (CI) (*p*-value)	1.548 (1.050–2.281) (0.027)	1.194 (0.842–1.693) (0.319)
SSTR3	HR (CI) (*p*-value)	1.008 (0.305–3.335) (0.990)	0.777 (0.257–2.345) (0.654)
SSTR4	HR (CI) (*p*-value)	1.761 (0.146–21.212) (0.656)	2.289 (0.246–21.343) (0.467)
SSTR5	HR (CI) (*p*-value)	1.172 (0.335–4.103) (0.804)	1.797 (0.574–5.628) (0.314)

Abbreviations: CI, confidence interval; HR, hazard ratio; n, events; OS, overall survival; TTR, time to recurrence.

## Data Availability

The data presented in this study are available on request from the corresponding author.

## Supplementary Materials

The following are available online at https://www.mdpi.com/article/10.3390/cancers13225704/s1, Supplementary File S1: Image analysis in ImageJ, Supplementary File S2: Calculation of Digital Score in R.
